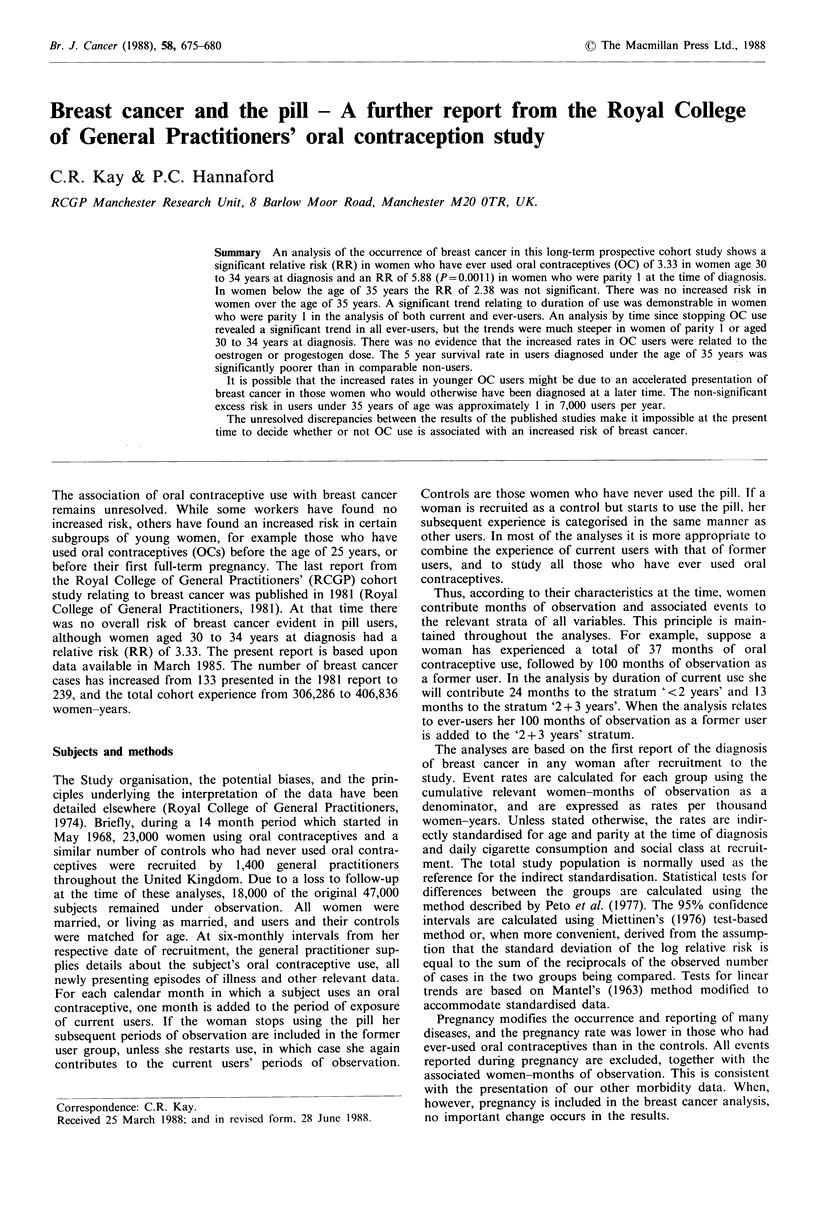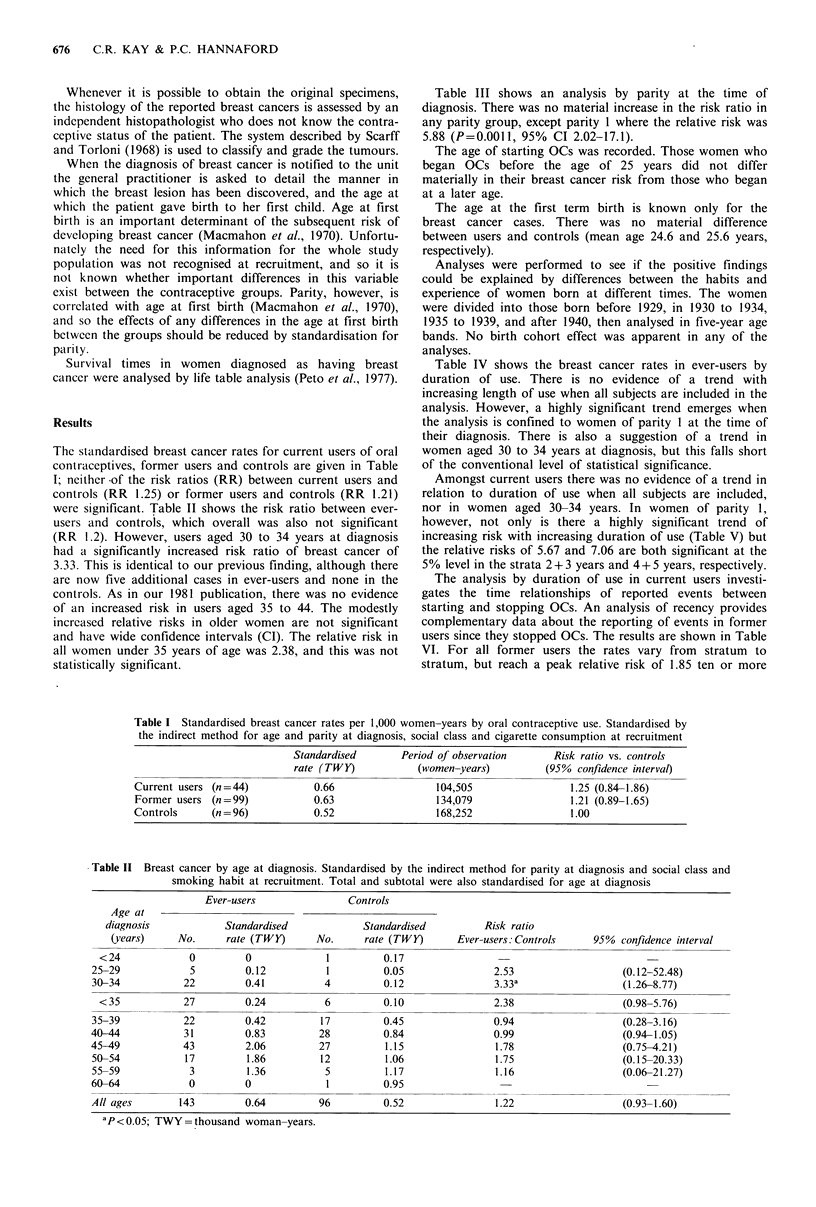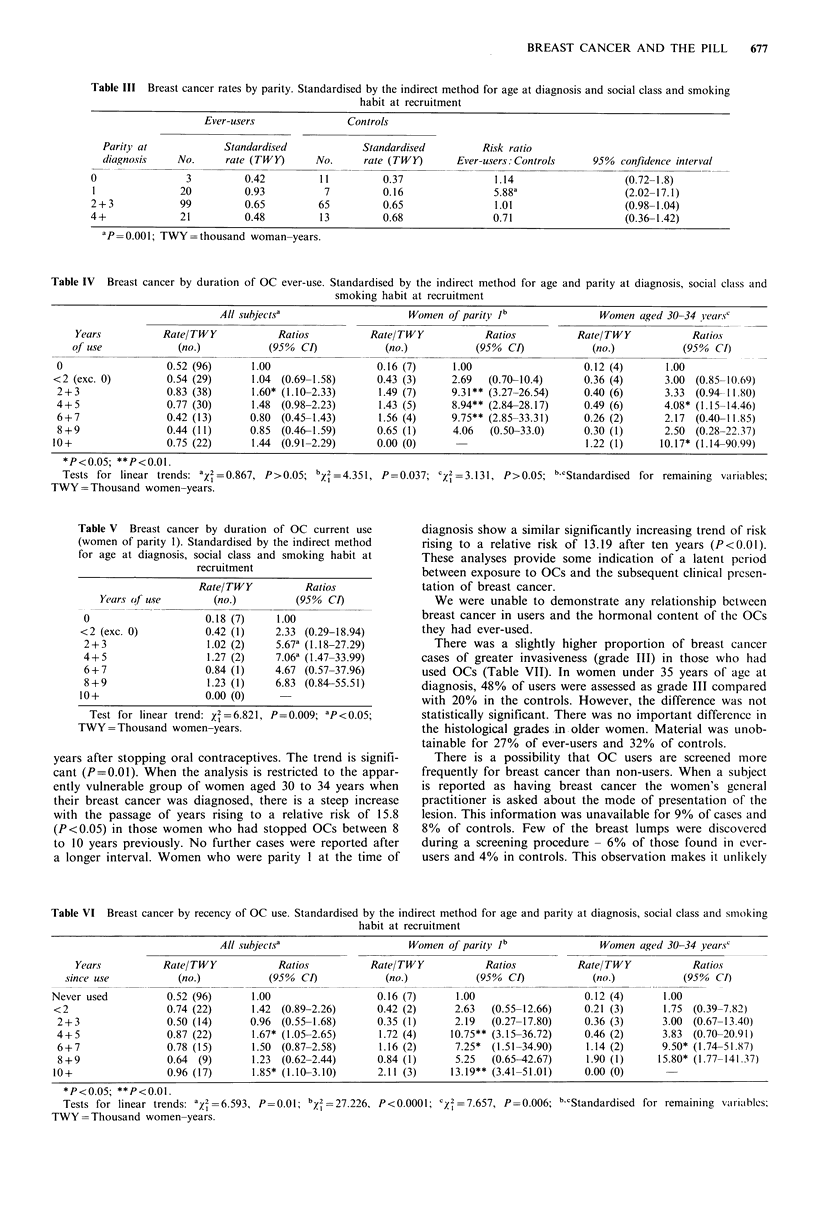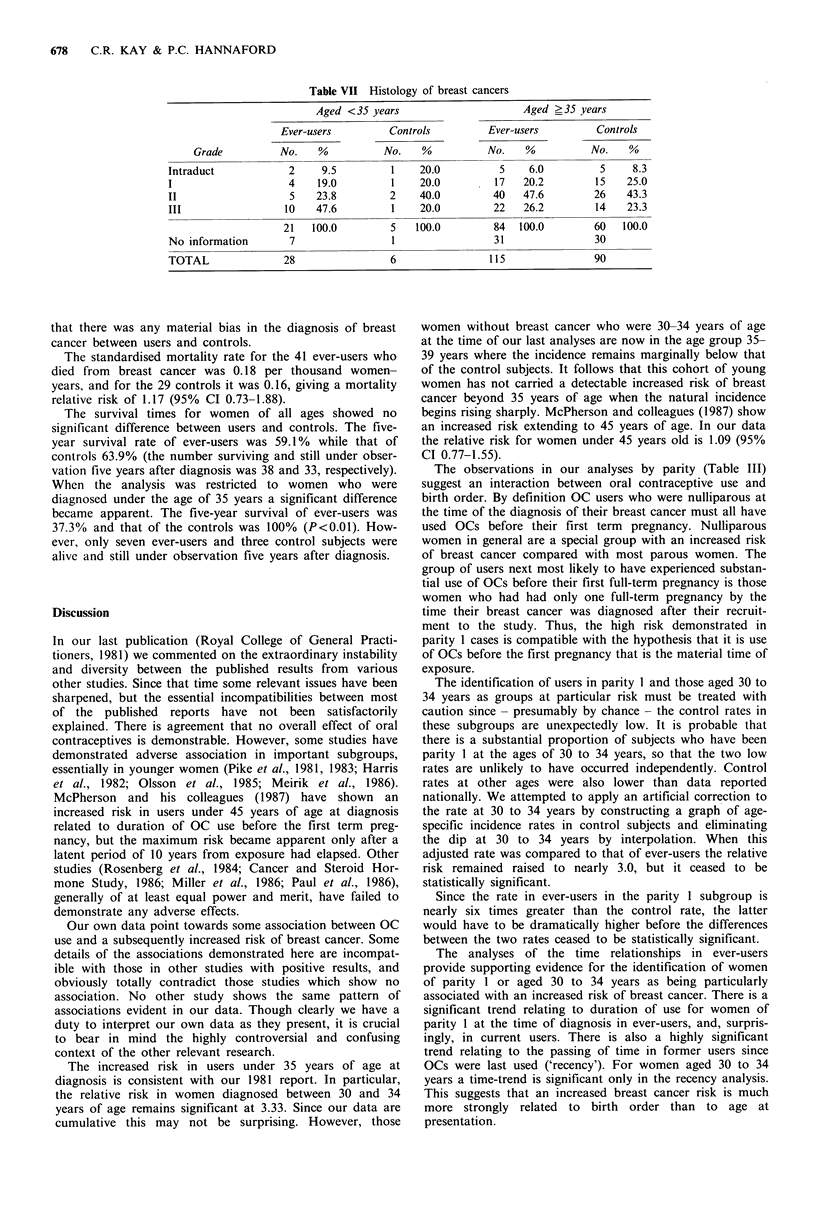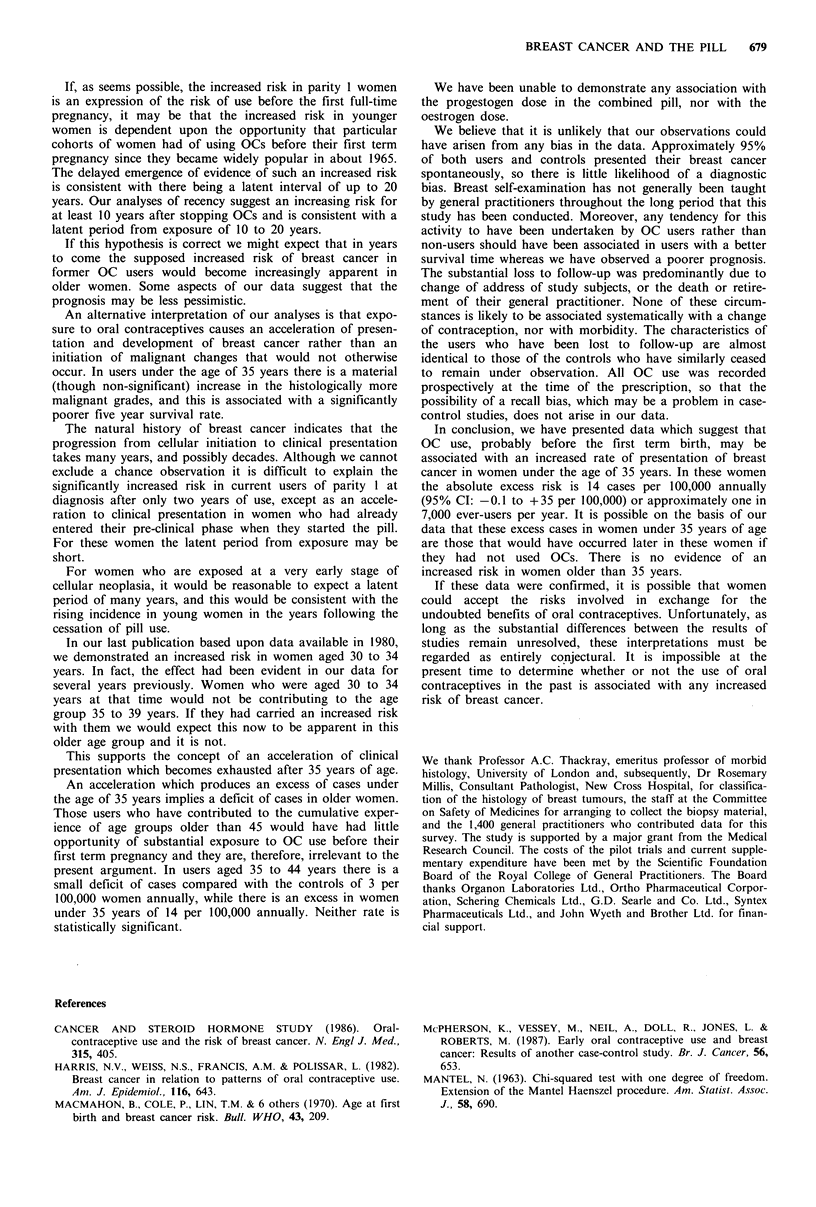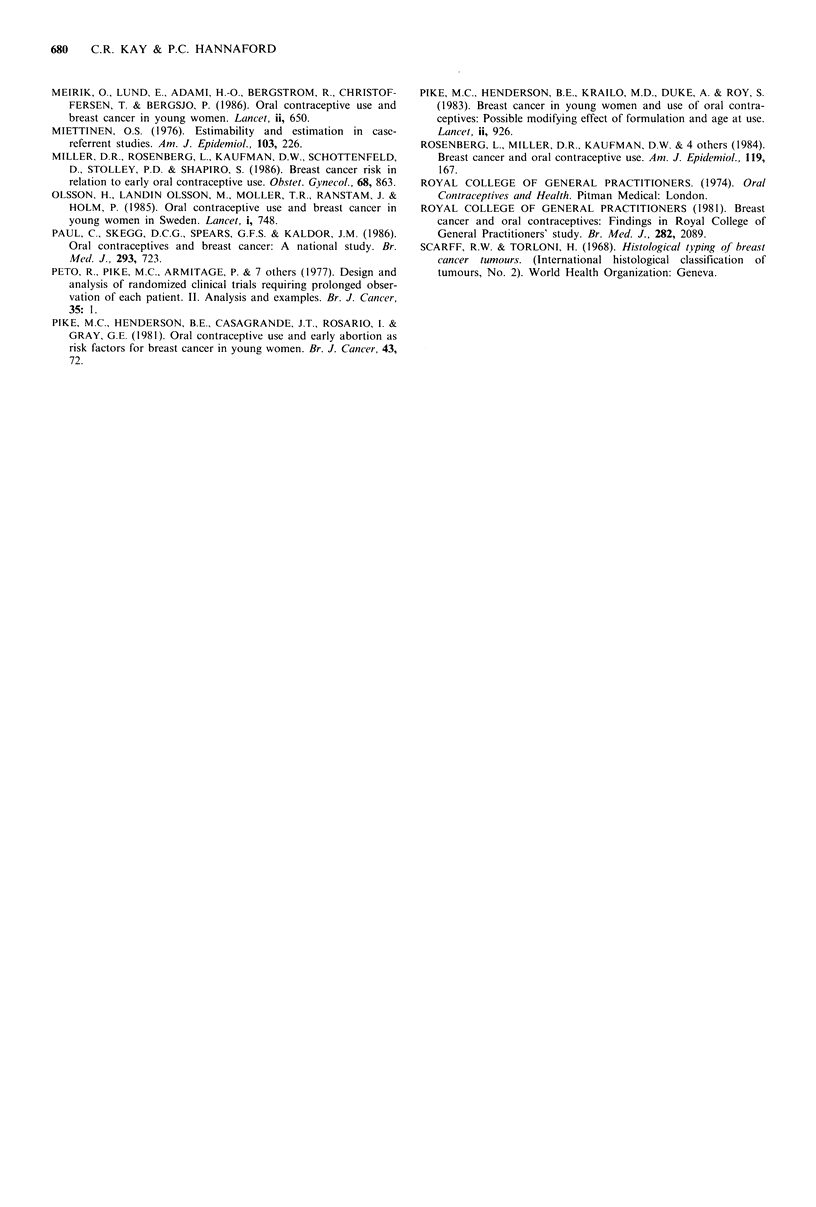# Breast cancer and the pill--a further report from the Royal College of General Practitioners' oral contraception study.

**DOI:** 10.1038/bjc.1988.285

**Published:** 1988-11

**Authors:** C. R. Kay, P. C. Hannaford

**Affiliations:** RCGP Manchester Research Unit, UK.

## Abstract

An analysis of the occurrence of breast cancer in this long-term prospective cohort study shows a significant relative risk (RR) in women who have ever used oral contraceptives (OC) of 3.33 in women age 30 to 34 years at diagnosis and an RR of 5.88 (P = 0.0011) in women who were parity 1 at the time of diagnosis. In women below the age of 35 years the RR of 2.38 was not significant. There was no increased risk in women over the age of 35 years. A significant trend relating to duration of use was demonstrable in women who were parity 1 in the analysis of both current and ever-users. An analysis by time since stopping OC use revealed a significant trend in all ever-users, but the trends were much steeper in women of parity 1 or aged 30 to 34 years at diagnosis. There was no evidence that the increased rates in OC users were related to the oestrogen or progestogen dose. The 5 year survival rate in users diagnosed under the age of 35 years was significantly poorer than in comparable non-users. It is possible that the increased rates in younger OC users might be due to an accelerated presentation of breast cancer in those women who would otherwise have been diagnosed at a later time. The non-significant excess risk in users under 35 years of age was approximately 1 in 7,000 users per year. The unresolved discrepancies between the results of the published studies make it impossible at the present time to decide whether or not OC use is associated with an increased risk of breast cancer.


Br. J. Cancer (1988), 58, 675–680 © The Macmillan Press Ltd., 1988

### Breast cancer and the pill – A further report from the Royal College of General Practitioners' oral contraception study

C.R. Kay & P.C. Hannaford

*RCGP Manchester Research Unit, 8 Barlow Moor Road, Manchester M20 0TR, UK.*

**Summary** An analysis of the occurrence of breast cancer in this long-term prospective cohort study shows a significant relative risk (RR) in women who have ever used oral contraceptives (OC) of 3.33 in women age 30 to 34 years at diagnosis and an RR of 5.88 ($P=0.0011$) in women who were parity 1 at the time of diagnosis. In women below the age of 35 years the RR of 2.38 was not significant. There was no increased risk in women over the age of 35 years. A significant trend relating to duration of use was demonstrable in women who were parity 1 in the analysis of both current and ever-users. An analysis by time since stopping OC use revealed a significant trend in all ever-users, but the trends were much steeper in women of parity 1 or aged 30 to 34 years at diagnosis. There was no evidence that the increased rates in OC users were related to the oestrogen or progestogen dose. The 5 year survival rate in users diagnosed under the age of 35 years was significantly poorer than in comparable non-users.

It is possible that the increased rates in younger OC users might be due to an accelerated presentation of breast cancer in those women who would otherwise have been diagnosed at a later time. The non-significant excess risk in users under 35 years of age was approximately 1 in 7,000 users per year.

The unresolved discrepancies between the results of the published studies make it impossible at the present time to decide whether or not OC use is associated with an increased risk of breast cancer.

The association of oral contraceptive use with breast cancer remains unresolved. While some workers have found no increased risk, others have found an increased risk in certain subgroups of young women, for example those who have used oral contraceptives (OCs) before the age of 25 years, or before their first full-term pregnancy. The last report from the Royal College of General Practitioners' (RCGP) cohort study relating to breast cancer was published in 1981 (Royal College of General Practitioners, 1981). At that time there was no overall risk of breast cancer evident in pill users, although women aged 30 to 34 years at diagnosis had a relative risk (RR) of 3.33. The present report is based upon data available in March 1985. The number of breast cancer cases has increased from 133 presented in the 1981 report to 239, and the total cohort experience from 306,286 to 406,836 women–years.

### Subjects and methods

The Study organisation, the potential biases, and the principles underlying the interpretation of the data have been detailed elsewhere (Royal College of General Practitioners, 1974). Briefly, during a 14 month period which started in May 1968, 23,000 women using oral contraceptives and a similar number of controls who had never used oral contraceptives were recruited by 1,400 general practitioners throughout the United Kingdom. Due to a loss to follow-up at the time of these analyses, 18,000 of the original 47,000 subjects remained under observation. All women were married, or living as married, and users and their controls were matched for age. At six-monthly intervals from her respective date of recruitment, the general practitioner supplies details about the subject's oral contraceptive use, all newly presenting episodes of illness and other relevant data. For each calendar month in which a subject uses an oral contraceptive, one month is added to the period of exposure of current users. If the woman stops using the pill her subsequent periods of observation are included in the former user group, unless she restarts use, in which case she again contributes to the current users' periods of observation. Controls are those women who have never used the pill. If a woman is recruited as a control but starts to use the pill, her subsequent experience is categorised in the same manner as other users. In most of the analyses it is more appropriate to combine the experience of current users with that of former users, and to study all those who have ever used oral contraceptives.

Thus, according to their characteristics at the time, women contribute months of observation and associated events to the relevant strata of all variables. This principle is maintained throughout the analyses. For example, suppose a woman has experienced a total of 37 months of oral contraceptive use, followed by 100 months of observation as a former user. In the analysis by duration of current use she will contribute 24 months to the stratum '<2 years' and 13 months to the stratum '2+3 years'. When the analysis relates to ever-users her 100 months of observation as a former user is added to the '2+3 years' stratum.

The analyses are based on the first report of the diagnosis of breast cancer in any woman after recruitment to the study. Event rates are calculated for each group using the cumulative relevant women–months of observation as a denominator, and are expressed as rates per thousand women–years. Unless stated otherwise, the rates are indirectly standardised for age and parity at the time of diagnosis and daily cigarette consumption and social class at recruitment. The total study population is normally used as the reference for the indirect standardisation. Statistical tests for differences between the groups are calculated using the method described by Peto *et al.* (1977). The 95% confidence intervals are calculated using Miettinen's (1976) test-based method or, when more convenient, derived from the assumption that the standard deviation of the log relative risk is equal to the sum of the reciprocals of the observed number of cases in the two groups being compared. Tests for linear trends are based on Mantel's (1963) method modified to accommodate standardised data.

Pregnancy modifies the occurrence and reporting of many diseases, and the pregnancy rate was lower in those who had ever-used oral contraceptives than in the controls. All events reported during pregnancy are excluded, together with the associated women–months of observation. This is consistent with the presentation of our other morbidity data. When, however, pregnancy is included in the breast cancer analysis, no important change occurs in the results.

Correspondence: C.R. Kay.
Received 25 March 1988; and in revised form, 28 June 1988.

Whenever it is possible to obtain the original specimens, the histology of the reported breast cancers is assessed by an independent histopathologist who does not know the contraceptive status of the patient. The system described by Scarff and Torloni (1968) is used to classify and grade the tumours.

When the diagnosis of breast cancer is notified to the unit the general practitioner is asked to detail the manner in which the breast lesion has been discovered, and the age at which the patient gave birth to her first child. Age at first birth is an important determinant of the subsequent risk of developing breast cancer (Macmahon *et al.*, 1970). Unfortunately the need for this information for the whole study population was not recognised at recruitment, and so it is not known whether important differences in this variable exist between the contraceptive groups. Parity, however, is correlated with age at first birth (Macmahon *et al.*, 1970), and so the effects of any differences in the age at first birth between the groups should be reduced by standardisation for parity.

Survival times in women diagnosed as having breast cancer were analysed by life table analysis (Peto *et al.*, 1977).

### Results

The standardised breast cancer rates for current users of oral contraceptives, former users and controls are given in Table I; neither of the risk ratios (RR) between current users and controls (RR 1.25) or former users and controls (RR 1.21) were significant. Table II shows the risk ratio between ever-users and controls, which overall was also not significant (RR 1.2). However, users aged 30 to 34 years at diagnosis had a significantly increased risk ratio of breast cancer of 3.33. This is identical to our previous finding, although there are now five additional cases in ever-users and none in the controls. As in our 1981 publication, there was no evidence of an increased risk in users aged 35 to 44. The modestly increased relative risks in older women are not significant and have wide confidence intervals (CI). The relative risk in all women under 35 years of age was 2.38, and this was not statistically significant.

Table III shows an analysis by parity at the time of diagnosis. There was no material increase in the risk ratio in any parity group, except parity 1 where the relative risk was 5.88 ($P=0.0011$, 95% CI 2.02–17.1).

The age of starting OCs was recorded. Those women who began OCs before the age of 25 years did not differ materially in their breast cancer risk from those who began at a later age.

The age at the first term birth is known only for the breast cancer cases. There was no material difference between users and controls (mean age 24.6 and 25.6 years, respectively).

Analyses were performed to see if the positive findings could be explained by differences between the habits and experience of women born at different times. The women were divided into those born before 1929, in 1930 to 1934, 1935 to 1939, and after 1940, then analysed in five-year age bands. No birth cohort effect was apparent in any of the analyses.

Table IV shows the breast cancer rates in ever-users by duration of use. There is no evidence of a trend with increasing length of use when all subjects are included in the analysis. However, a highly significant trend emerges when the analysis is confined to women of parity 1 at the time of their diagnosis. There is also a suggestion of a trend in women aged 30 to 34 years at diagnosis, but this falls short of the conventional level of statistical significance.

Amongst current users there was no evidence of a trend in relation to duration of use when all subjects are included, nor in women aged 30–34 years. In women of parity 1, however, not only is there a highly significant trend of increasing risk with increasing duration of use (Table V) but the relative risks of 5.67 and 7.06 are both significant at the 5% level in the strata 2+3 years and 4+5 years, respectively.

The analysis by duration of use in current users investigates the time relationships of reported events between starting and stopping OCs. An analysis of recency provides complementary data about the reporting of events in former users since they stopped OCs. The results are shown in Table VI. For all former users the rates vary from stratum to stratum, but reach a peak relative risk of 1.85 ten or more

**Table I** Standardised breast cancer rates per 1,000 women-years by oral contraceptive use. Standardised by the indirect method for age and parity at diagnosis, social class and cigarette consumption at recruitment

| | *Standardised rate (TWY)* | *Period of observation (women-years)* | *Risk ratio* vs. *controls (95% confidence interval)* |
|---|---|---|---|
| Current users ($n=44$) | 0.66 | 104,505 | 1.25 (0.84–1.86) |
| Former users ($n=99$) | 0.63 | 134,079 | 1.21 (0.89–1.65) |
| Controls ($n=96$) | 0.52 | 168,252 | 1.00 |

**Table II** Breast cancer by age at diagnosis. Standardised by the indirect method for parity at diagnosis and social class and smoking habit at recruitment. Total and subtotal were also standardised for age at diagnosis

| *Age at diagnosis (years)* | *Ever-users* No. | *Ever-users* Standardised rate (TWY) | *Controls* No. | *Controls* Standardised rate (TWY) | *Risk ratio Ever-users:Controls* | *95% confidence interval* |
|---|---|---|---|---|---|---|
| <24 | 0 | 0 | 1 | 0.17 | — | — |
| 25–29 | 5 | 0.12 | 1 | 0.05 | 2.53 | (0.12–52.48) |
| 30–34 | 22 | 0.41 | 4 | 0.12 | 3.33[a] | (1.26–8.77) |
| <35 | 27 | 0.24 | 6 | 0.10 | 2.38 | (0.98–5.76) |
| 35–39 | 22 | 0.42 | 17 | 0.45 | 0.94 | (0.28–3.16) |
| 40–44 | 31 | 0.83 | 28 | 0.84 | 0.99 | (0.94–1.05) |
| 45–49 | 43 | 2.06 | 27 | 1.15 | 1.78 | (0.75–4.21) |
| 50–54 | 17 | 1.86 | 12 | 1.06 | 1.75 | (0.15–20.33) |
| 55–59 | 3 | 1.36 | 5 | 1.17 | 1.16 | (0.06–21.27) |
| 60–64 | 0 | 0 | 1 | 0.95 | — | — |
| *All ages* | 143 | 0.64 | 96 | 0.52 | 1.22 | (0.93–1.60) |

[a] $P<0.05$; TWY = thousand woman-years.

**Table III** Breast cancer rates by parity. Standardised by the indirect method for age at diagnosis and social class and smoking habit at recruitment

| | *Ever-users* | | *Controls* | | | |
|---|---|---|---|---|---|---|
| *Parity at diagnosis* | *No.* | *Standardised rate (TWY)* | *No.* | *Standardised rate (TWY)* | *Risk ratio Ever-users: Controls* | *95% confidence interval* |
| 0 | 3 | 0.42 | 11 | 0.37 | 1.14 | (0.72–1.8) |
| 1 | 20 | 0.93 | 7 | 0.16 | 5.88[a] | (2.02–17.1) |
| 2+3 | 99 | 0.65 | 65 | 0.65 | 1.01 | (0.98–1.04) |
| 4+ | 21 | 0.48 | 13 | 0.68 | 0.71 | (0.36–1.42) |

[a]$P=0.001$; TWY = thousand woman-years.

**Table IV** Breast cancer by duration of OC ever-use. Standardised by the indirect method for age and parity at diagnosis, social class and smoking habit at recruitment

| | *All subjects*[a] | | *Women of parity 1*[b] | | *Women aged 30–34 years*[c] | |
|---|---|---|---|---|---|---|
| *Years of use* | *Rate/TWY (no.)* | *Ratios (95% CI)* | *Rate/TWY (no.)* | *Ratios (95% CI)* | *Rate/TWY (no.)* | *Ratios (95% CI)* |
| 0 | 0.52 (96) | 1.00 | 0.16 (7) | 1.00 | 0.12 (4) | 1.00 |
| <2 (exc. 0) | 0.54 (29) | 1.04 (0.69–1.58) | 0.43 (3) | 2.69 (0.70–10.4) | 0.36 (4) | 3.00 (0.85–10.69) |
| 2+3 | 0.83 (38) | 1.60* (1.10–2.33) | 1.49 (7) | 9.31** (3.27–26.54) | 0.40 (6) | 3.33 (0.94–11.80) |
| 4+5 | 0.77 (30) | 1.48 (0.98–2.23) | 1.43 (5) | 8.94** (2.84–28.17) | 0.49 (6) | 4.08* (1.15–14.46) |
| 6+7 | 0.42 (13) | 0.80 (0.45–1.43) | 1.56 (4) | 9.75** (2.85–33.31) | 0.26 (2) | 2.17 (0.40–11.85) |
| 8+9 | 0.44 (11) | 0.85 (0.46–1.59) | 0.65 (1) | 4.06 (0.50–33.0) | 0.30 (1) | 2.50 (0.28–22.37) |
| 10+ | 0.75 (22) | 1.44 (0.91–2.29) | 0.00 (0) | — | 1.22 (1) | 10.17* (1.14–90.99) |

*$P<0.05$; **$P<0.01$.
Tests for linear trends: [a]$\chi_1^2=0.867$, $P>0.05$; [b]$\chi_1^2=4.351$, $P=0.037$; [c]$\chi_1^2=3.131$, $P>0.05$; [b,c]Standardised for remaining variables; TWY = Thousand women-years.

**Table V** Breast cancer by duration of OC current use (women of parity 1). Standardised by the indirect method for age at diagnosis, social class and smoking habit at recruitment

| *Years of use* | *Rate/TWY (no.)* | *Ratios (95% CI)* |
|---|---|---|
| 0 | 0.18 (7) | 1.00 |
| <2 (exc. 0) | 0.42 (1) | 2.33 (0.29–18.94) |
| 2+3 | 1.02 (2) | 5.67[a] (1.18–27.29) |
| 4+5 | 1.27 (2) | 7.06[a] (1.47–33.99) |
| 6+7 | 0.84 (1) | 4.67 (0.57–37.96) |
| 8+9 | 1.23 (1) | 6.83 (0.84–55.51) |
| 10+ | 0.00 (0) | — |

Test for linear trend: $\chi_1^2=6.821$, $P=0.009$; [a]$P<0.05$; TWY = Thousand women-years.

years after stopping oral contraceptives. The trend is significant ($P=0.01$). When the analysis is restricted to the apparently vulnerable group of women aged 30 to 34 years when their breast cancer was diagnosed, there is a steep increase with the passage of years rising to a relative risk of 15.8 ($P<0.05$) in those women who had stopped OCs between 8 to 10 years previously. No further cases were reported after a longer interval. Women who were parity 1 at the time of diagnosis show a similar significantly increasing trend of risk rising to a relative risk of 13.19 after ten years ($P<0.01$). These analyses provide some indication of a latent period between exposure to OCs and the subsequent clinical presentation of breast cancer.

We were unable to demonstrate any relationship between breast cancer in users and the hormonal content of the OCs they had ever-used.

There was a slightly higher proportion of breast cancer cases of greater invasiveness (grade III) in those who had used OCs (Table VII). In women under 35 years of age at diagnosis, 48% of users were assessed as grade III compared with 20% in the controls. However, the difference was not statistically significant. There was no important difference in the histological grades in older women. Material was unobtainable for 27% of ever-users and 32% of controls.

There is a possibility that OC users are screened more frequently for breast cancer than non-users. When a subject is reported as having breast cancer the women's general practitioner is asked about the mode of presentation of the lesion. This information was unavailable for 9% of cases and 8% of controls. Few of the breast lumps were discovered during a screening procedure – 6% of those found in ever-users and 4% in controls. This observation makes it unlikely

**Table VI** Breast cancer by recency of OC use. Standardised by the indirect method for age and parity at diagnosis, social class and smoking habit at recruitment

| | *All subjects*[a] | | *Women of parity 1*[b] | | *Women aged 30–34 years*[c] | |
|---|---|---|---|---|---|---|
| *Years since use* | *Rate/TWY (no.)* | *Ratios (95% CI)* | *Rate/TWY (no.)* | *Ratios (95% CI)* | *Rate/TWY (no.)* | *Ratios (95% CI)* |
| Never used | 0.52 (96) | 1.00 | 0.16 (7) | 1.00 | 0.12 (4) | 1.00 |
| <2 | 0.74 (22) | 1.42 (0.89–2.26) | 0.42 (2) | 2.63 (0.55–12.66) | 0.21 (3) | 1.75 (0.39–7.82) |
| 2+3 | 0.50 (14) | 0.96 (0.55–1.68) | 0.35 (1) | 2.19 (0.27–17.80) | 0.36 (3) | 3.00 (0.67–13.40) |
| 4+5 | 0.87 (22) | 1.67* (1.05–2.65) | 1.72 (4) | 10.75** (3.15–36.72) | 0.46 (2) | 3.83 (0.70–20.91) |
| 6+7 | 0.78 (15) | 1.50 (0.87–2.58) | 1.16 (2) | 7.25* (1.51–34.90) | 1.14 (2) | 9.50* (1.74–51.87) |
| 8+9 | 0.64 (9) | 1.23 (0.62–2.44) | 0.84 (1) | 5.25 (0.65–42.67) | 1.90 (1) | 15.80* (1.77–141.37) |
| 10+ | 0.96 (17) | 1.85* (1.10–3.10) | 2.11 (3) | 13.19** (3.41–51.01) | 0.00 (0) | — |

*$P<0.05$; **$P<0.01$.

Tests for linear trends: [a]$\chi_1^2=6.593$, $P=0.01$; [b]$\chi_1^2=27.226$, $P<0.0001$; [c]$\chi_1^2=7.657$, $P=0.006$; [b,c]Standardised for remaining variables; TWY = Thousand women-years.

**Table VII** Histology of breast cancers

| Grade | Aged <35 years | | | | Aged ≥35 years | | | |
|---|---|---|---|---|---|---|---|---|
| | Ever-users | | Controls | | Ever-users | | Controls | |
| | No. | % | No. | % | No. | % | No. | % |
| Intraduct | 2 | 9.5 | 1 | 20.0 | 5 | 6.0 | 5 | 8.3 |
| I | 4 | 19.0 | 1 | 20.0 | 17 | 20.2 | 15 | 25.0 |
| II | 5 | 23.8 | 2 | 40.0 | 40 | 47.6 | 26 | 43.3 |
| III | 10 | 47.6 | 1 | 20.0 | 22 | 26.2 | 14 | 23.3 |
| | 21 | 100.0 | 5 | 100.0 | 84 | 100.0 | 60 | 100.0 |
| No information | 7 | | 1 | | 31 | | 30 | |
| TOTAL | 28 | | 6 | | 115 | | 90 | |

that there was any material bias in the diagnosis of breast cancer between users and controls.

The standardised mortality rate for the 41 ever-users who died from breast cancer was 0.18 per thousand women–years, and for the 29 controls it was 0.16, giving a mortality relative risk of 1.17 (95% CI 0.73–1.88).

The survival times for women of all ages showed no significant difference between users and controls. The five-year survival rate of ever-users was 59.1% while that of controls 63.9% (the number surviving and still under observation five years after diagnosis was 38 and 33, respectively). When the analysis was restricted to women who were diagnosed under the age of 35 years a significant difference became apparent. The five-year survival of ever-users was 37.3% and that of the controls was 100% ($P<0.01$). However, only seven ever-users and three control subjects were alive and still under observation five years after diagnosis.

### Discussion

In our last publication (Royal College of General Practitioners, 1981) we commented on the extraordinary instability and diversity between the published results from various other studies. Since that time some relevant issues have been sharpened, but the essential incompatibilities between most of the published reports have not been satisfactorily explained. There is agreement that no overall effect of oral contraceptives is demonstrable. However, some studies have demonstrated adverse association in important subgroups, essentially in younger women (Pike *et al.*, 1981, 1983; Harris *et al.*, 1982; Olsson *et al.*, 1985; Meirik *et al.*, 1986). McPherson and his colleagues (1987) have shown an increased risk in users under 45 years of age at diagnosis related to duration of OC use before the first term pregnancy, but the maximum risk became apparent only after a latent period of 10 years from exposure had elapsed. Other studies (Rosenberg *et al.*, 1984; Cancer and Steroid Hormone Study, 1986; Miller *et al.*, 1986; Paul *et al.*, 1986), generally of at least equal power and merit, have failed to demonstrate any adverse effects.

Our own data point towards some association between OC use and a subsequently increased risk of breast cancer. Some details of the associations demonstrated here are incompatible with those in other studies with positive results, and obviously totally contradict those studies which show no association. No other study shows the same pattern of associations evident in our data. Though clearly we have a duty to interpret our own data as they present, it is crucial to bear in mind the highly controversial and confusing context of the other relevant research.

The increased risk in users under 35 years of age at diagnosis is consistent with our 1981 report. In particular, the relative risk in women diagnosed between 30 and 34 years of age remains significant at 3.33. Since our data are cumulative this may not be surprising. However, those women without breast cancer who were 30–34 years of age at the time of our last analyses are now in the age group 35–39 years where the incidence remains marginally below that of the control subjects. It follows that this cohort of young women has not carried a detectable increased risk of breast cancer beyond 35 years of age when the natural incidence begins rising sharply. McPherson and colleagues (1987) show an increased risk extending to 45 years of age. In our data the relative risk for women under 45 years old is 1.09 (95% CI 0.77–1.55).

The observations in our analyses by parity (Table III) suggest an interaction between oral contraceptive use and birth order. By definition OC users who were nulliparous at the time of the diagnosis of their breast cancer must all have used OCs before their first term pregnancy. Nulliparous women in general are a special group with an increased risk of breast cancer compared with most parous women. The group of users next most likely to have experienced substantial use of OCs before their first full-term pregnancy is those women who had had only one full-term pregnancy by the time their breast cancer was diagnosed after their recruitment to the study. Thus, the high risk demonstrated in parity 1 cases is compatible with the hypothesis that it is use of OCs before the first pregnancy that is the material time of exposure.

The identification of users in parity 1 and those aged 30 to 34 years as groups at particular risk must be treated with caution since – presumably by chance – the control rates in these subgroups are unexpectedly low. It is probable that there is a substantial proportion of subjects who have been parity 1 at the ages of 30 to 34 years, so that the two low rates are unlikely to have occurred independently. Control rates at other ages were also lower than data reported nationally. We attempted to apply an artificial correction to the rate at 30 to 34 years by constructing a graph of age-specific incidence rates in control subjects and eliminating the dip at 30 to 34 years by interpolation. When this adjusted rate was compared to that of ever-users the relative risk remained raised to nearly 3.0, but it ceased to be statistically significant.

Since the rate in ever-users in the parity 1 subgroup is nearly six times greater than the control rate, the latter would have to be dramatically higher before the differences between the two rates ceased to be statistically significant.

The analyses of the time relationships in ever-users provide supporting evidence for the identification of women of parity 1 or aged 30 to 34 years as being particularly associated with an increased risk of breast cancer. There is a significant trend relating to duration of use for women of parity 1 at the time of diagnosis in ever-users, and, surprisingly, in current users. There is also a highly significant trend relating to the passing of time in former users since OCs were last used ('recency'). For women aged 30 to 34 years a time-trend is significant only in the recency analysis. This suggests that an increased breast cancer risk is much more strongly related to birth order than to age at presentation.

If, as seems possible, the increased risk in parity 1 women is an expression of the risk of use before the first full-time pregnancy, it may be that the increased risk in younger women is dependent upon the opportunity that particular cohorts of women had of using OCs before their first term pregnancy since they became widely popular in about 1965. The delayed emergence of evidence of such an increased risk is consistent with there being a latent interval of up to 20 years. Our analyses of recency suggest an increasing risk for at least 10 years after stopping OCs and is consistent with a latent period from exposure of 10 to 20 years.

If this hypothesis is correct we might expect that in years to come the supposed increased risk of breast cancer in former OC users would become increasingly apparent in older women. Some aspects of our data suggest that the prognosis may be less pessimistic.

An alternative interpretation of our analyses is that exposure to oral contraceptives causes an acceleration of presentation and development of breast cancer rather than an initiation of malignant changes that would not otherwise occur. In users under the age of 35 years there is a material (though non-significant) increase in the histologically more malignant grades, and this is associated with a significantly poorer five year survival rate.

The natural history of breast cancer indicates that the progression from cellular initiation to clinical presentation takes many years, and possibly decades. Although we cannot exclude a chance observation it is difficult to explain the significantly increased risk in current users of parity 1 at diagnosis after only two years of use, except as an acceleration to clinical presentation in women who had already entered their pre-clinical phase when they started the pill. For these women the latent period from exposure may be short.

For women who are exposed at a very early stage of cellular neoplasia, it would be reasonable to expect a latent period of many years, and this would be consistent with the rising incidence in young women in the years following the cessation of pill use.

In our last publication based upon data available in 1980, we demonstrated an increased risk in women aged 30 to 34 years. In fact, the effect had been evident in our data for several years previously. Women who were aged 30 to 34 years at that time would not be contributing to the age group 35 to 39 years. If they had carried an increased risk with them we would expect this now to be apparent in this older age group and it is not.

This supports the concept of an acceleration of clinical presentation which becomes exhausted after 35 years of age.

An acceleration which produces an excess of cases under the age of 35 years implies a deficit of cases in older women. Those users who have contributed to the cumulative experience of age groups older than 45 would have had little opportunity of substantial exposure to OC use before their first term pregnancy and they are, therefore, irrelevant to the present argument. In users aged 35 to 44 years there is a small deficit of cases compared with the controls of 3 per 100,000 women annually, while there is an excess in women under 35 years of 14 per 100,000 annually. Neither rate is statistically significant.

We have been unable to demonstrate any association with the progestogen dose in the combined pill, nor with the oestrogen dose.

We believe that it is unlikely that our observations could have arisen from any bias in the data. Approximately 95% of both users and controls presented their breast cancer spontaneously, so there is little likelihood of a diagnostic bias. Breast self-examination has not generally been taught by general practitioners throughout the long period that this study has been conducted. Moreover, any tendency for this activity to have been undertaken by OC users rather than non-users should have been associated in users with a better survival time whereas we have observed a poorer prognosis. The substantial loss to follow-up was predominantly due to change of address of study subjects, or the death or retirement of their general practitioner. None of these circumstances is likely to be associated systematically with a change of contraception, nor with morbidity. The characteristics of the users who have been lost to follow-up are almost identical to those of the controls who have similarly ceased to remain under observation. All OC use was recorded prospectively at the time of the prescription, so that the possibility of a recall bias, which may be a problem in case-control studies, does not arise in our data.

In conclusion, we have presented data which suggest that OC use, probably before the first term birth, may be associated with an increased rate of presentation of breast cancer in women under the age of 35 years. In these women the absolute excess risk is 14 cases per 100,000 annually (95% CI: −0.1 to +35 per 100,000) or approximately one in 7,000 ever-users per year. It is possible on the basis of our data that these excess cases in women under 35 years of age are those that would have occurred later in these women if they had not used OCs. There is no evidence of an increased risk in women older than 35 years.

If these data were confirmed, it is possible that women could accept the risks involved in exchange for the undoubted benefits of oral contraceptives. Unfortunately, as long as the substantial differences between the results of studies remain unresolved, these interpretations must be regarded as entirely conjectural. It is impossible at the present time to determine whether or not the use of oral contraceptives in the past is associated with any increased risk of breast cancer.

We thank Professor A.C. Thackray, emeritus professor of morbid histology, University of London and, subsequently, Dr Rosemary Millis, Consultant Pathologist, New Cross Hospital, for classification of the histology of breast tumours, the staff at the Committee on Safety of Medicines for arranging to collect the biopsy material, and the 1,400 general practitioners who contributed data for this survey. The study is supported by a major grant from the Medical Research Council. The costs of the pilot trials and current supplementary expenditure have been met by the Scientific Foundation Board of the Royal College of General Practitioners. The Board thanks Organon Laboratories Ltd., Ortho Pharmaceutical Corporation, Schering Chemicals Ltd., G.D. Searle and Co. Ltd., Syntex Pharmaceuticals Ltd., and John Wyeth and Brother Ltd. for financial support.

### References

CANCER AND STEROID HORMONE STUDY (1986). Oral-contraceptive use and the risk of breast cancer. *N. Engl J. Med.*, **315**, 405.

HARRIS, N.V., WEISS, N.S., FRANCIS, A.M. & POLISSAR, L. (1982). Breast cancer in relation to patterns of oral contraceptive use. *Am. J. Epidemiol.*, **116**, 643.

MACMAHON, B., COLE, P., LIN, T.M. & 6 others (1970). Age at first birth and breast cancer risk. *Bull. WHO*, **43**, 209.

McPHERSON, K., VESSEY, M., NEIL, A., DOLL, R., JONES, L. & ROBERTS, M. (1987). Early oral contraceptive use and breast cancer: Results of another case-control study. *Br. J. Cancer*, **56**, 653.

MANTEL, N. (1963). Chi-squared test with one degree of freedom. Extension of the Mantel Haenszel procedure. *Am. Statist. Assoc. J.*, **58**, 690.

MEIRIK, O., LUND, E., ADAMI, H.-O., BERGSTROM, R., CHRISTOFFERSEN, T. & BERGSJO, P. (1986). Oral contraceptive use and breast cancer in young women. *Lancet*, **ii**, 650.

MIETTINEN, O.S. (1976). Estimability and estimation in case-referrent studies. *Am. J. Epidemiol.*, **103**, 226.

MILLER, D.R., ROSENBERG, L., KAUFMAN, D.W., SCHOTTENFELD, D., STOLLEY, P.D. & SHAPIRO, S. (1986). Breast cancer risk in relation to early oral contraceptive use. *Obstet. Gynecol.*, **68**, 863.

OLSSON, H., LANDIN OLSSON, M., MOLLER, T.R., RANSTAM, J. & HOLM, P. (1985). Oral contraceptive use and breast cancer in young women in Sweden. *Lancet*, **i**, 748.

PAUL, C., SKEGG, D.C.G., SPEARS, G.F.S. & KALDOR, J.M. (1986). Oral contraceptives and breast cancer: A national study. *Br. Med. J.*, **293**, 723.

PETO, R., PIKE, M.C., ARMITAGE, P. & 7 others (1977). Design and analysis of randomized clinical trials requiring prolonged observation of each patient. II. Analysis and examples. *Br. J. Cancer*, **35**: 1.

PIKE, M.C., HENDERSON, B.E., CASAGRANDE, J.T., ROSARIO, I. & GRAY, G.E. (1981). Oral contraceptive use and early abortion as risk factors for breast cancer in young women. *Br. J. Cancer*, **43**, 72.

PIKE, M.C., HENDERSON, B.E., KRAILO, M.D., DUKE, A. & ROY, S. (1983). Breast cancer in young women and use of oral contraceptives: Possible modifying effect of formulation and age at use. *Lancet*, **ii**, 926.

ROSENBERG, L., MILLER, D.R., KAUFMAN, D.W. & 4 others (1984). Breast cancer and oral contraceptive use. *Am. J. Epidemiol.*, **119**, 167.

ROYAL COLLEGE OF GENERAL PRACTITIONERS. (1974). *Oral Contraceptives and Health*. Pitman Medical: London.

ROYAL COLLEGE OF GENERAL PRACTITIONERS (1981). Breast cancer and oral contraceptives: Findings in Royal College of General Practitioners' study. *Br. Med. J.*, **282**, 2089.

SCARFF, R.W. & TORLONI, H. (1968). *Histological typing of breast cancer tumours*. (International histological classification of tumours, No. 2). World Health Organization: Geneva.